# Association between Glucose Levels and Intraocular Pressure: Pre- and Postprandial Analysis in Diabetic and Nondiabetic Patients

**DOI:** 10.1155/2015/832058

**Published:** 2015-01-06

**Authors:** Luis Guilherme Milesi Pimentel, Carolina P. B. Gracitelli, Leticia Sant'Ana Cardoso da Silva, Aline Katia Siqueira Souza, Tiago Santos Prata

**Affiliations:** ^1^Department of Ophthalmology, Federal University of São Paulo, 04021-001 Vila Mariana, SP, Brazil; ^2^Glaucoma Unit, Hospital Medicina dos Olhos, 06018-180 Osasco, SP, Brazil

## Abstract

The aim of this study was to evaluate the relationship between glucose levels and intraocular pressure (IOP) fluctuation in diabetic and nondiabetic patients. Seventeen nondiabetic and 20 diabetic subjects underwent a complete ophthalmic examination, capillary glucose testing, and applanation tonometry in two distinct situations: first, fasting for at least 8 hours and, second, postprandial measurements. Baseline glucose levels were higher in diabetic patients (*P* < 0.001). Postprandial IOP was significantly higher than baseline IOP in diabetic (*P* < 0.001) and nondiabetic patients (*P* = 0.006). Postprandial glucose levels were significantly higher than baseline measurements in both diabetic (*P* = 0.005) and nondiabetic patients (*P* = 0.015). There was a significant association between glucose levels variation and IOP change in both diabetic patients (*R* ^2^ = 0.540; *P* < 0.001) and nondiabetic individuals (*R* ^2^ = 0.291; *P* = 0.025). There is also a significant association between the baseline glucose levels and IOP change in diabetic group (*R* ^2^ = 0.445; *P* = 0.001). In a multivariable model, the magnitude of glucose level change remained significantly associated with IOP variation even including age, baseline IOP, ancestry, and gender as a confounding factor (*P* < 0.001). We concluded that there is a significant association between blood glucose levels and IOP variation, especially in diabetic patients.

## 1. Introduction

Glaucoma is an optic neuropathy characterized by progressive degeneration of retinal ganglion cells (RGCs) and their axons, resulting in changes in the appearance of the optic disc and visual field loss [1]. Although glaucoma is a multifactorial disease, elevated intraocular pressure (IOP) remains its major known risk factor [2, 3]. Several large randomized clinical trials underscored the relationship between IOP and glaucoma development and progression [2–6]. Therefore, adequate determination of an individual IOP value is of utmost importance in the management of the disease.

The IOP can be influenced by different systemic factors such as hypertension [7–9], atherosclerotic diseases [7], body mass index [10], and diabetes [7, 11, 12]. For instance, Lee and colleagues studying the relationship between IOP and systemic disorders found that increased mean blood pressure is strongly correlated with risk of increased IOP.

Although diabetes is associated with higher IOP values in most population studies, the underlying mechanisms are still unclear [7, 11, 12]. Recent studies have suggested that changes in corneal biomechanics (increased corneal hysteresis) in diabetic eyes would lead to overestimated IOP measurements [13–15]. However, it is not known whether variations in glucose levels could lead to IOP changes in diabetic and nondiabetic individuals. As diabetes and glaucoma (or ocular hypertension) coexist in many patients, a better understanding about how variations in glucose levels can affect IOP changes would give additional information to the IOP assessment.

Therefore, we sought to determine the relationship between glucose levels variation and IOP fluctuation in diabetic and nondiabetic patients.

## 2. Methods

This prospective observational study adhered to the tenets of the Declaration of Helsinki and was approved by the Institutional Review Board of the Federal University of São Paulo. In addition, written informed consent was obtained from all participants.

### 2.1. Patients

We prospectively enrolled diabetic patients and nondiabetic individuals. All participants underwent a complete ophthalmological examination including review of medical history, best-corrected visual acuity, slit-lamp biomicroscopy, IOP measurement, gonioscopy, dilated funduscopic examination, and refraction. Exclusion criteria were glaucoma diagnosis or ocular hypertension, corneal opacity or irregularities that could alter the ophthalmological examination, refractive error greater than ±5 D spherical or cylindrical greater than ±3 D, and central corneal thickness (based on ultrasound pachymetry) above 600 microns or below 450 microns. Diabetes was defined according to self-reported physician diagnosis, and all diabetic patients were under medical treatment. Healthy subjects were recruited from the general population or from spouses and relatives of diabetic patients. They were defined as self-reported history of normal glucose level in the past two years.

### 2.2. Capillary Glucose Testing

All participants underwent capillary glucose testing in two distinct situations: first, baseline measurements (fasting for exactly 8 hours, i.e., after overnight fasting) and, second, postprandial measurements (exactly 2 hours after the meal, i.e., after lunch time). The same examiner performed all measurements in a masked fashion. The measurement of capillary glucose was performed by collecting blood from the patient's finger, pierced through the skin by a lancet and checked with an automated device (OneTouch LifeScan, Johnson & Johnson, CA, USA).

### 2.3. Intraocular Pressure Assessment

Immediately after the capillary glucose testing, IOP was measured in both eyes (i.e., fasting for exactly 8 hours and exactly 2 hours after lunch time) of each patient by Goldmann tonometry applanation (Haag-Streit, Köniz, Switzerland). The calibration of each instrument was checked at the beginning of each session, according to the manufacturers' instructions [16]. All measurements were taken with the patient in a sitting position. The same examiner performed all IOP measurements in a masked fashion and a different examiner performed the glucose levels measurements. Whenever both eyes were eligible, the right eye was arbitrarily chosen for analysis.

### 2.4. Statistical Analysis

Descriptive statistics included mean and standard deviation values for normally distributed variables. We used skewness/kurtosis tests and histograms to check normality. Paired *t*-test was used for comparison of IOP values between each time point (baseline and postprandial). For variables whose distribution rejected normality, we used a nonparametric test (Wilcoxon rank-sum test).

The association between changes in glucose levels and IOP variation was investigated using univariable and multivariable regression analyses (including age and baseline IOP). The baseline glucose level was not included in the multivariable model to avoid collinearity between glucose level variations and baseline glucose level. Whenever both eyes were eligible, the right eye was arbitrarily chosen for this analysis.

All statistical analyses were performed with commercially available software (Stata, version 13; StataCorp LP, College Station, TX, USA). The *α* level (type I error) was set at 0.05.

## 3. Results

A total of 37 patients (17 nondiabetic and 20 diabetic) were included. Among those diabetics, 5 had type 1 (all of them were insulin dependent) and 15 had type 2 diabetes (all of them were not insulin dependent). Diabetic patients had been followed for an average of 13.7 ± 10.2 years. Age and IOP did not differ significantly between the two groups (*P* = 0.182). Baseline glucose levels were higher in the group of diabetic patients (*P* < 0.001). Demographic and clinical data of each group are provided in detail in Table 1.

**Figure 1:**
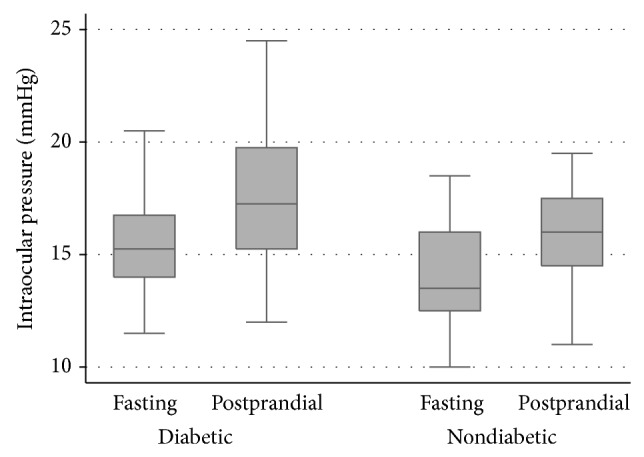
Box plots showing the distribution of average IOP in diabetic and nondiabetic groups in the two different times of measurements. Box represents median and interquartile range. Whiskers correspond to maximum and minimum 1.5 IQR.

Postprandial IOP was significantly higher than baseline IOP in diabetic (17.8 ± 0.80 versus 15.5 ± 0.55 mmHg; *P* < 0.001) and nondiabetic patients (15.9 ± 0.77 versus 14.3 ± 0.72 mmHg; *P* = 0.006).  Figure 1 shows the IOP distribution of the two groups at each time point. Postprandial glucose levels were significantly higher than baseline measurements in both diabetic (mean increase of 62 mg/dL; *P* = 0.005) and nondiabetic patients (mean increase of 31.5 mg/dL; *P* = 0.015).

In the univariable analysis, there is a significant (positive) association between glucose levels variation and IOP change in both diabetic (*R* ^2^ = 0.540; *P* < 0.001) and nondiabetic patients (*R* ^2^ = 0.291; *P* = 0.025). There is also a significant (positive) association between the baseline glucose levels (fasting glucose levels) and IOP change in diabetic group (*R* ^2^ = 0.445; *P* = 0.001); however this association was not found in nondiabetic group (*R* ^2^ = 0.142; *P* = 0.136).

**Figure 2:**
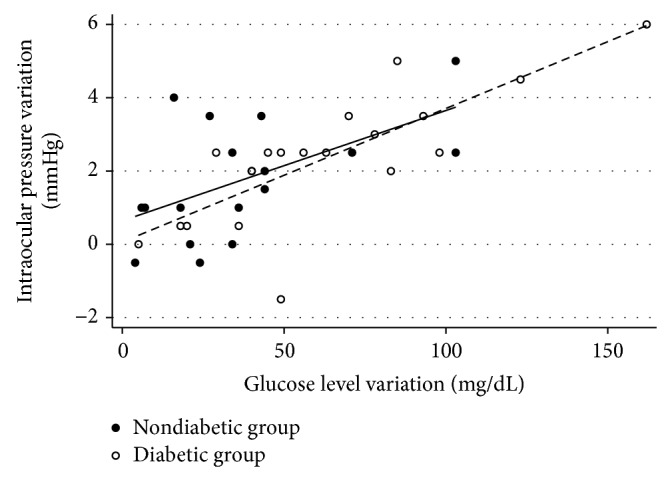
Scatter plot showing the association between IOP variation and glucose levels variation in both groups.

In the multivariable analysis, the magnitude of glucose level change remained significantly associated with IOP variation (*P* < 0.001) even including age, baseline IOP, ancestry, and gender as confounding factors.  Figure 2 shows the association between glucose levels variation and IOP change in each group.

**Figure 3:**
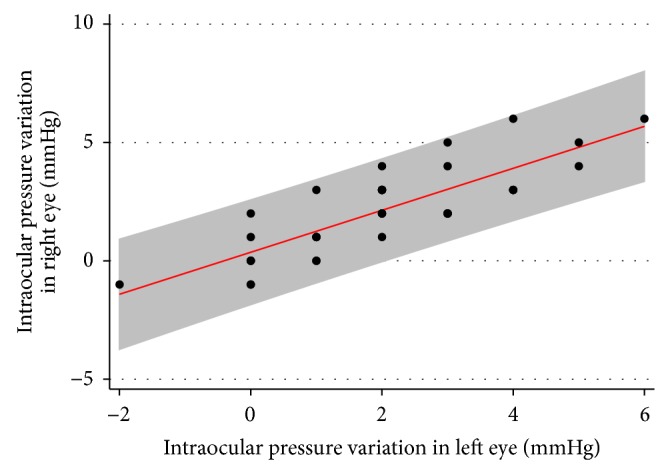
Scatter plot depicting the relationship between IOP variations in the right and in the left eye (shaded area represents 95% confidence interval of the regression).

In addition, there was a strong correlation between the IOP variation in right eye and IOP variation in the left eye (*R* ^2^ = 0.826).  Figure 3 illustrates the relationship between IOP variations in the right and in the left eye.

## 4. Discussion

Although many patients referred for an ophthalmological examination have diabetes, until now it was not known whether blood glucose levels could influence or not an individual IOP variation. To the best of our knowledge, this is the first report that provides evidence that glucose levels are significantly associated with IOP changes not only in diabetic patients but in healthy individuals as well. We found a significant increase in postprandial IOP values in both groups, which seem to be explained in part by the magnitude of glucose levels variation in these patients.

The relationship between diabetes and IOP has been underscored in previous publications. In sum, their results reveal a positive association between diabetes and IOP [12, 17–21]. Evaluating factors possibly associated with IOP in a black population, the Barbados Eye Study documented that the presence of diabetes, among other factors such as systolic blood pressure and age, was positively correlated with higher IOP values [18]. Moreover, in the Blue Mountain Eye Study, by exploring the relationship between diabetes and open-angle glaucoma, the authors found that glaucoma prevalence was higher in diabetic patients compared to those without diabetes (5.5% versus 2.8%, OR = 2.12) [12]. Although the above-mentioned studies did not evaluate the association between blood glucose levels variation and IOP changes specifically, we believe that our findings indirectly corroborate their results.

Regarding the association between glucose levels and IOP, there are scant data in the literature. Larsen and colleagues [22] found lower IOP values during severe hypoglycemia. In addition, Traisman et al. [23] and associates, while assessing IOP in patients with blood glucose values under and above 200 mg/dL, observed higher IOP values in those with glucose levels above 200 mg/dL (mean difference of 1.3 mmHg). Unfortunately, none of these studies sought to determine the association between the magnitude of glucose levels variation and IOP change in diabetic and nondiabetic patients, which hinders a straight comparison with our findings. Nevertheless, we believe that our data are in agreement with these two latter studies, as we found a mean IOP increase of 2.3 and 1.6 mmHg in diabetic and nondiabetic patients, respectively, during the postprandial period. Finally, the discrepancy in the literature results could be explained in part by the differences in studies designs and populations and possibly by the influence of other systemic associations such as hypertension, obesity, and other conditions that were not evaluated.

Several hypotheses have been created to explain the association between high glucose levels and IOP. Some researchers believe that there are genetic factors associated in family history of diabetes [24]. Other researchers agree with the idea that a diabetic person could have an autonomic dysfunction which would lead to an IOP increase [25]. However some authors believe that elevated blood glucose results in the induction of an osmotic gradient which leads to fluid shifts into the intraocular space [12].

At this point, we believe that it is important to discuss the main clinical implications of our findings. Ophthalmologists often see diabetic patients on daily practice. Many of these diabetic patients already have glaucoma (or ocular hypertension) or are glaucoma suspects. Although most attention is given to each 1 mmHg variation in IOP, the glycemic control is rarely taken into account. Based on our findings, glycemic levels variation may influence IOP change and is therefore relevant for diagnosis and treatment management, especially in diabetic patients, whose average IOP variation (between baseline and postprandial measurements) was approximately 15% (for an average glycemic variation of 40%). The fact that we also documented a significant association in nondiabetic individuals makes the influence of glycemic levels on IOP even more relevant. As such, we believe that clinicians should consider the patient's glycemic status and glucose level variations concurrently with IOP values assessment in certain cases, especially in diabetic patients using, for example, peripheral capillary blood as a screening for glucose level variations.

It is important to stress and discuss some specific characteristics and limitations of the present study. First, it is limited by its small sample size; however, even with a small sample we found a significant association between blood glucose levels and IOP variation, especially in diabetic patients (20 patients). Second, glucose levels were assessed solely twice (baseline and postprandial). Multiple measurements would have provided additional data and possibly allowed a more detailed analysis about the association between glucose variation and IOP change. Third, the investigation of other systemic comorbidities by means of questionnaire may have been insufficient. Fourth, we did not correlate the duration of diabetes and IOP. However, it is important to emphasize that previously published data indicate that whereas the duration of the diabetic disease is an important parameter for the incidence and severity of retinopathy, there is no such influence when it comes to IOP [26, 27]. Lastly, we used peripheral capillary blood for the glucose analysis. This method is acceptable for patient self-monitoring or screening purposes. However, different studies showed evidences that peripheral capillary blood and venous (antecubital fossa) blood samples could be comparable and can have similar influence after meal [28]. In addition, further studies should be done to evaluate the causative relationship between glucose levels and IOP variation. Our findings suggest that there is an association between blood glucose levels and IOP values; however, IOP variation could have been affected by different factors other than glucose levels. Therefore, longitudinal studies should help us to better understand the connection between these two variations. Furthermore, another relevant factor that could be addressed in future studies is the corneal hysteresis that could be measured by Ocular Response Analyzer (Reichert Ophthalmic Instruments, Depew, NY, USA). Different studies have reported that corneal hysteresis is affected by HbA1c, intraocular pressure, and central corneal thickness [14].

## 5. Conclusions

In conclusion, our results suggest that there is a significant association between blood glucose levels and IOP values, especially in diabetic patients. Postprandial IOP seems to be significantly higher in these patients compared to baseline values, revealing a strong association with the magnitude of glucose level increase. This fact should be considered while assessing IOP values and fluctuation especially in diabetic patients.

## Figures and Tables

**Table 1:** Demographic and clinical variables in eyes of nondiabetics and diabetics group.

	Nondiabetic patients (*N* = 17)	Diabetic patients (*N* = 20)	*P* value
Age (±SD), years^a^	55.2 ± 18.2	61.0 ± 9.9	0.230^b^
Ancestry, %			0.858^d^
European	8 (47.1%)	10 (50%)	
African	9 (52.9%)	10 (50%)	
Gender, %			0.769^d^
Female	11 (64.7%)	12 (60.0%)	
Male	6 (35.3%)	8 (40.0%)	
VA, log⁡MAR	0.06 ± 0.7	0.05 ± 0.08	0.590^c^
CCT, *μ*m	519.8 ± 18.2	516.2 ± 18.2	0.568^c^

VA = visual acuity; CCT = central cornea thickness; SD = standard deviation.

^a^Mean (±SD).

^b^ *t*-test.

^c^Wilcoxon rank-sum test.

^d^Pearson chi-square test.
